# Effect of the Affordable Medicines Facility for malaria (AMFm) on the availability of antimalarials in Nigeria

**DOI:** 10.5281/zenodo.10870095

**Published:** 2015-05-09

**Authors:** Arinola Joda, Nnenna Ezeigwe, Lilian Oguguo, Ogori Taylor, Godwin Ntadom

**Affiliations:** 1 Department of Clinical Pharmacy and Biopharmacy, Faculty of Pharmacy, University of Lagos, Idiaraba Campus, Idiaraba, Lagos, Nigeria; 2 National Malaria Elimination Program, Federal Ministry of Health, Abuja, Nigeria; 3 World Health Organization (WHO), Abuja, Nigeria

## Abstract

**Background:**

Malaria is one of the most important causes of mortality worldwide. Use of the most effective treatments remains inadequate for those in need and there is concern over the emergence of resistance. Rapid, accurate and accessible detection of malaria parasites plays a role in promoting more rational use of increasingly costly drugs in many endemic areas. Rapid diagnostic tests (RDTs) offer the potential to provide accurate diagnosis to all at-risk populations for the first time, reaching those unable to access good quality microscopy services. In 2010, the Global Fund launched the Affordable Medicines Facility for malaria (AMFm) designed to increase access and use of good quality artemisinin-based combination therapies (ACTs) for malaria treatment. AMFm involves manufacturer price negotiations, subsidies and other interventions. The aim of this study was to document the availability of ACTs and RDTs provided under the National Malaria Elimination Programme via the AMFm financing strategy.

**Materials and Methods:**

Investigators were systematically selected and trained on the data collection tool from the World Health Organization/Health Action International (WHO/HAI) Workbook. Data was collected from public and private facilities in 12 states in the six geopolitical zones of Nigeria in April and May 2014. Returned survey forms were checked, entered and verified. Data analysis was carried out using the embedded analysis toolkit in the WHO/HAI Workbook after double-entry and auto-checking of data. Data was analysed for the public and private sectors.

**Results:**

Seven AMFm products are available in the country, and include AL (IPCA), Artemef (Cipla), Coartem AMFm (Novartis), Combisunate (Ajanta), Lumartem (Cipla) as well as Arsuamoon (Guilin) and Coarsucam (Sanofi-Winthrop). The results reveal that antimalarials are largely concentrated in the private sector (private pharmacies and PPMVs). About 86% of the surveyed facilities had at least one AMFm AL product whereas only 18% had any AMFm AA product. Results show that the availability of the various AMFm AL products varies across the country. Lumartem by Cipla has the highest national availability with 26.4%, closely followed by AL (IPCA) with 25.7%. Twenty seven (%) of the facilities had an RDT in stock.

**Conclusion:**

The results obtained in this survey show that continuous monitoring of the antimalarial drug landscape is required to track progress in the fight against malaria in the country.

## 1 Introduction

Malaria remains an important cause of death and illness in children and adults in Africa [1-3], although various reports indicate growing evidence for a substantial decline in malaria transmission, morbidity and mortality over the last decade [4,5]. Other reports, however, document that malaria mortality has risen in recent years, probably due to increasing resistance to antimalarial medicines [6-8]. In 2012, an estimated 207 million cases and 627,000 deaths were recorded globally with most of the cases occurring in sub-Saharan Africa [9]. More than 80% of estimated malaria deaths in 2012 occurred in just 17 countries, and 80% of cases occur in 18 countries, with the Democratic Republic of Congo and Nigeria together accounting for 40% of the estimated global total [9,10].

Malaria control requires an integrated approach comprising prevention including vector control and treatment with effective antimalarials [9,11,12]. The affordable and widely available antimalarial chloroquine that was a mainstay of malaria control in the past is now ineffective in most *P. falciparum* malaria-endemic areas, and resistance to sulfadoxine-pyrimethamine (SP) is increasing rapidly [6,13]. The discovery and development of the artemisinin derivatives in China, and their evaluation in Southeast Asia and other regions provided a new class of highly effective antimalarials, and have already transformed the chemotherapy of malaria in many African countries [6,14,15]. Artemisinin-based combination therapies (ACTs) are now generally considered the best current treatment for uncomplicated falciparum malaria [2,10,14]. In Nigeria, Artemether/Lumefantrine (AL) and Artesunate/Amodiaquine (AA) were adopted in the revised malaria treatment policy as the drugs of choice for uncomplicated malaria [16].

The Affordable Medicines Facility−malaria (AMFm) is a financing mechanism run by the Global Fund to Fight AIDS, TB and Malaria [17,18]. This Fund is designed to expand access to the best treatments for malaria [2,17]. AMFm donors subsidise the price of ACTs in the public and private sectors by negotiating a reduced price for ACTs with manufacturers. Donors pay the majority of the reduced price of ACTs to manufacturers, lowering the cost to first-line buyers. First-line buyers purchase ACTs direct from manufacturers. Since first-line buyers pay a lower price for ACTs, consumers should also pay a lower price, making ACTs more broadly available and affordable [2,17,19]. In 2009, the baseline outlet survey carried out in Nigeria revealed that first line quality assured ACTs were available in 46% of the facilities surveyed [20].

Malaria rapid diagnostic tests or dipsticks assist in the diagnosis of malaria by showing evidence of the presence of malaria parasites in the blood. RDTs are an alternative to diagnosis based on clinical grounds or microscopy, particularly where good quality microscopy services cannot be readily accessed [21,22]. Effective use of RDTs will ensure rational use of antimalarial medicines as opposed to the current climate of presumptively treating all febrile cases for malaria [23,24].

The aim of this study was to document the availability of ACTs and RDTs provided under the National Malaria Elimination Program of the Federal Ministry of Health via the Affordable Medicines Facility for malaria financing strategy.

## 2 Materials and Methods

### 2.1 Location, target population and data collection

This survey, being a national survey, required that each of the geopolitical zones in the country was represented. There are six geopolitical zones in the country. Two states were randomly selected per geopolitical zone to provide 12 states for the study. Each state has three senatorial districts (Central, North and South Senatorial Districts) and outlets were selected from each of these districts in the selected states according to the sampling plan below (Table 1). The sampling plan was exceeded in many tertiary/secondary hospitals and pharmacies. The selected states were: Benue, FCT Abuja (North-Central, NC), Bauchi, Yobe (Northeast, NE), Kaduna, Kano (Northwest, NW), Abia, Anambra (Southeast, SE), Cross River, Rivers (South-South, SS) and Lagos and Oyo (Southwest, SW).

**Table 1.** Summary of the sampling of outlets in each state

Type of facility	Sampling plan	Total number per state	Sampling plan for outlets	Actual number of outlets analysed
***Public sector***				
Tertiary hospital	1 per state	1	12	18
Secondary hospital	1 per SD*	3	36	47
PHC	6 per SD	18	216	120
***Subtotal for public sector***		**22**	**264**	**185**
***Private sector***				
Pharmacy	2 per SD	6	24	77
Proprietary and Patent Medicine Vendors’	5 per SD	15	180	120
License (PPMVL) holders				
***Subtotal for private sector***		**21**	**206**	**197**
**Total number of facilities per state in the public and private sectors**		**43**	**470**	**386**

*: SD=Senatorial Districts

Surveys were conducted in both the private and public sector. In the public sector tertiary and secondary hospitals and primary healthcare (PHC) facilities were surveyed, whereas in the private sector community pharmacies (pharmacies) and premises of proprietary and patent medicine vendors’ licensees (PPMVLs) were accessed (Table 1).

Three data collectors (i.e. one data collector per senatorial district) resident in the state were recruited to be responsible for the quarterly monitoring activity. The data collectors were pharmacists recruited from the public and private sectors to obtain required information from the different facility types.

### 2.2 Research instrument

The WHO/HAI Essential Medicines workbook [25] was adapted for this survey. The survey form was adapted for local use and pretested in one state that was not involved in the actual survey. Data on availability of antimalarials and RDTs was collected in a sample of outlets in the public and private sector as shown (Table 1). However, the sampling plan for PHCs and PPMVLs greatly exceeded the number of records that can be entered into the WHO/ HAI Workbook. Surveys were performed according to a standardised protocol. In each outlet, data was collected on available AMFm antimalarials as well as other antimalarials found in the facility during the survey. The unit price of the available antimalarials was documented as the index of availability [26]. Upon finishing the field survey, a double input of data into the WHO/HAI Essential Medicines Workbook software was performed in order to reduce errors, and the Workbook’s auto checker was used in the verification process [25].

### 2.3 Data analysis

All collected data from public hospitals (tertiary and secondary) and private pharmacies (community pharmacies) was entered into the Workbook. For PHCs and PPMVLs, four survey forms in the Central Senatorial District along with each from those North and South of this were entered, resulting in a total of 10 outlets per state. This was necessary in order not to exceed the maximum number of records available in the WHO/HAI workbook (Table 1). Availability was expressed as the number of facilities with antimalarials on the day of data collection.

## 3 Results

### 3.1 National availability of antimalarials

**Figure 1.**
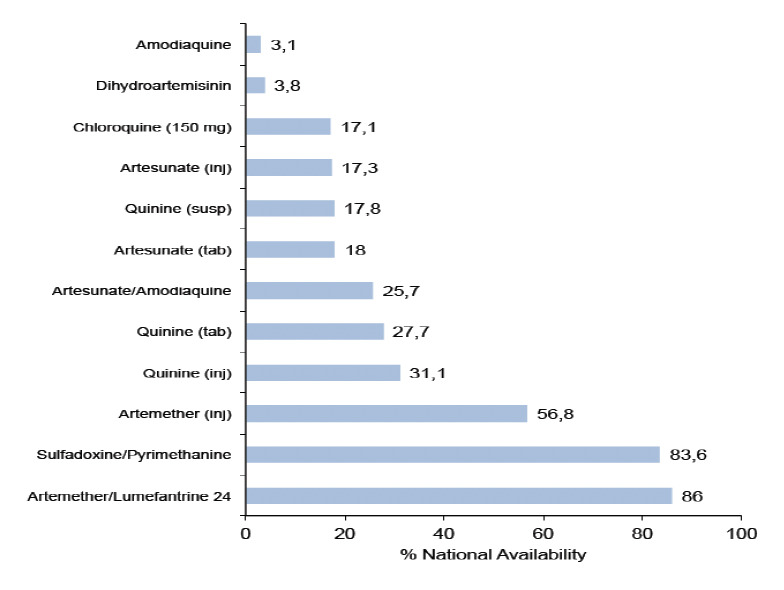
Availability of antimalarials (%) in Nigeria

Availability of the AMFm products varied considerably across the country and sectors. Nationally, the average availability of any AMFm AL product was 86%, whereas the corresponding value for any AMFm AA was 17.8%. Artemisinin monotherapy availability was 57% for artemether injection, 26% for artemisinin tablets, 17% for artesunate injection and 4% for dihydroartemisinin (DHA). Non-artemisinin therapy with SP was still high at 84%, low for chloroquine at 17% and very low for amodiaquine tablets at 3% (Fig. 1).

**Figure 2.**
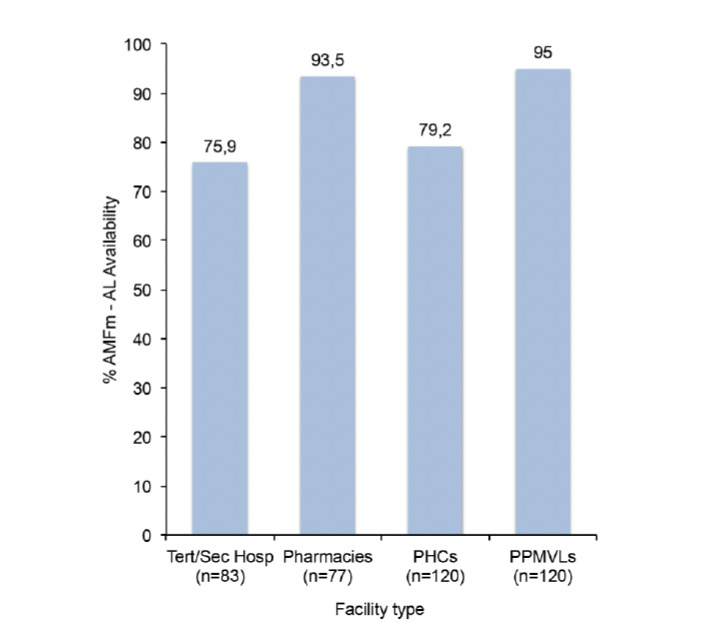
Availability of AMFm products by facility type. Tert/sec Hosp = tertiary/secondary hospital

**Figure 3.**
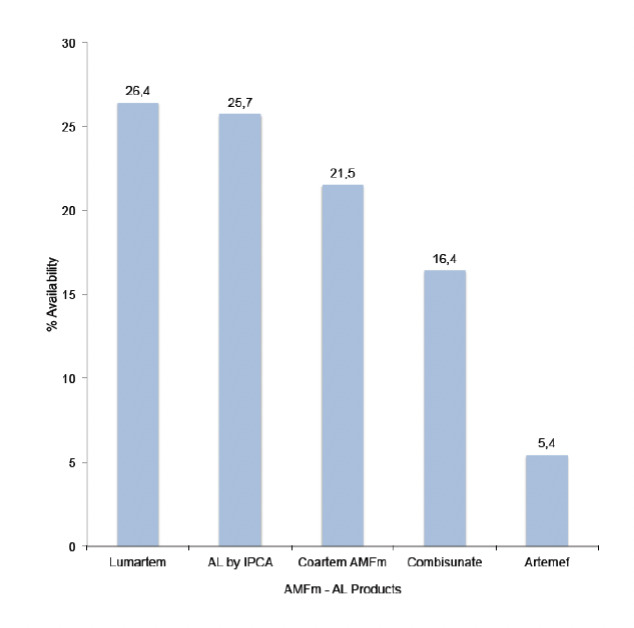
National availability (%) of AMFm AL products

The result shows that the private sector (pharmacies and PPMVLs) had the highest availability of AMFm AL products, with 94% of them having at least one product in stock on the day of the survey. By facility type, availability was 76%, 94%, 79% and 95% in the tertiary/secondary hospitals, pharmacies, PHCs and PPMVLs, respectively (Fig. 2). Seven AMFm products are available in the country subdivided into five Artemether/Lumefantrine products and two Artesunate/Amodiaquine products. The Artemether/Lumefantrine products are AL by Ipca, Artemef by Cipla, Coartem AMFm by Novartis, Combisunate by Ajanta and Lumartem by Cipla, whereas the Artesunate/Amodiaquine products are Arsuamoon by Guilin and Coarsucam by Sanofi-Winthrop. Of the Artemether/Lumefantrine AMFm products, Lumartem and AL by Ipca had the highest availability, representing approximately 26% of the AMFm AL products found. Artemef had the lowest representation at about 5% (Fig. 3). The Artesunate/Amodiaquine AMFm products were much less available than the Artemether/Lumefantrine AMFm products, with Coarsucam having 8% availability and Arsuamoon having only 2% availability.

### 3.2 Zonal availability of AMFm antimalarial products

**Figure 4.**
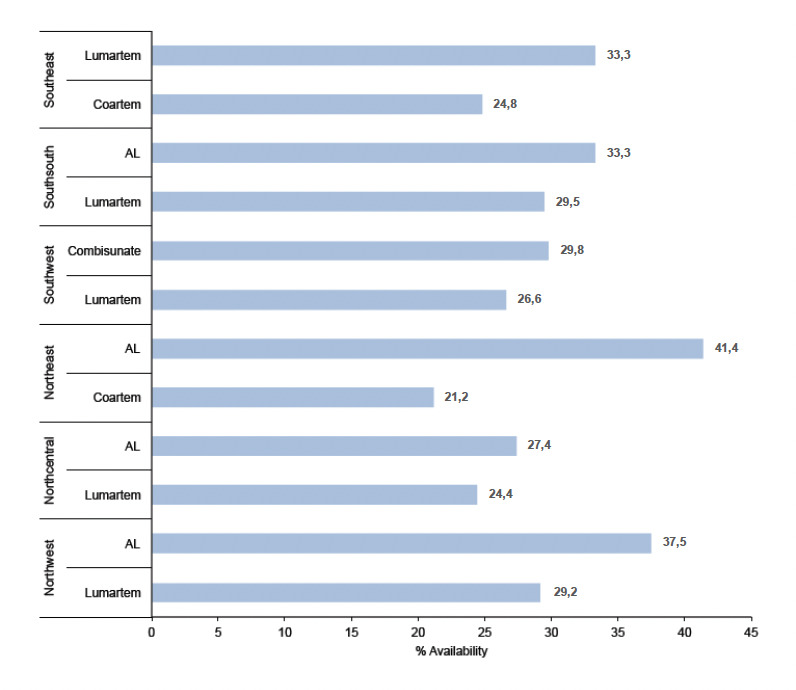
Two most available AMFm AL products by geographical zone

Zonal availability of AMFm products show that in the SE, SS, SW, NE, NC and NW Lumartem, AL (Ipca), Combisunate, AL (Ipca), AL (Ipca) and AL (Ipca) were most frequently available. Figure 4 shows the top two AMFm products available by geopolitical zone.

### 3.3 Rapid diagnostic test (RDT) availability

**Figure 5.**
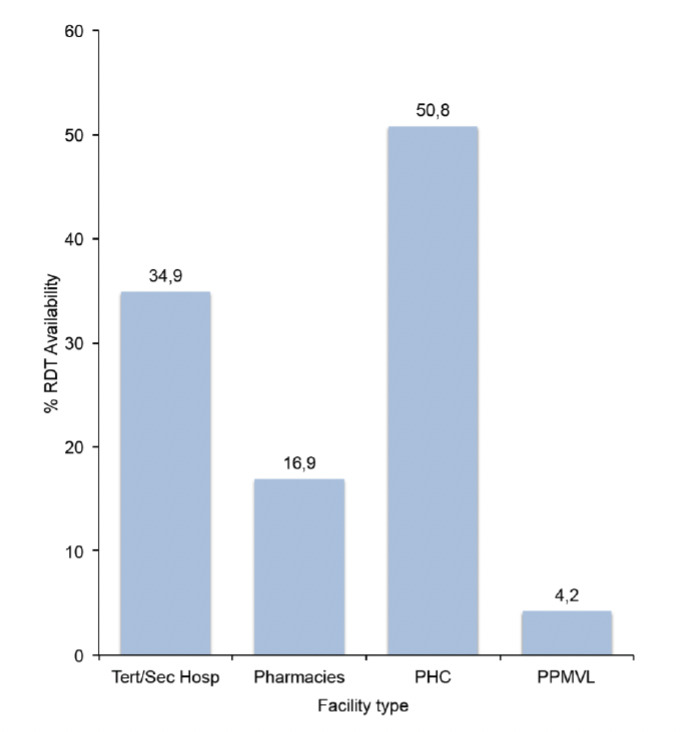
RDT availability (%) by facility type

**Figure 6.**
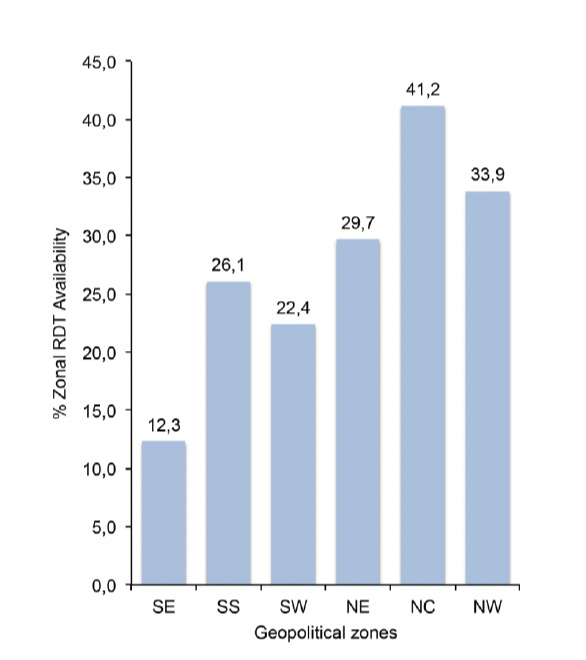
RDT availability (%) by geographical zone

Nationally, average RDT availability was 27%, with the public sector (tertiary/secondary hospitals and PHCs) having higher availability rates than the private sector (Pharmacies and PPMVLs). Slightly more than half of the PHCs had RDTs available at the time of the survey (Fig. 5). Zonal availability shows that the North Central zone had the highest availability of RDTs among the six geopolitical zones of the country (Fig. 6).

## 4 Discussion

The Affordable Medicines Facility for malaria (AMFm) put in place a bold financing plan for ACT availability covering half the population at risk of malaria in Africa [17]. Surveys are conducted at intervals to document the viability of the project and track progress.

### 4.1 National availability of antimalarials

AMFm increased availability and kept prices low, meeting its initial, ambitious benchmarks in most settings [17,20,27,28]. AMFm aims to increase the availability and use of ACTs by reducing their cost and thereby driving oral artemisinin monotherapies, which should only be used as part of combination therapies, or poor-quality antimalarial drugs from the market [17]. This survey shows that the availability of any AMFm Artemether/Lumefantrine has improved over the values obtained previously with a steady improvement from 46% in 2009 [20] to 54% in 2011 [2,29] and 86% achieved in this survey. In a soon-to-be-published outlet survey conducted in Nigeria in 2012, AMFm AL availability was found to be 91% [30].

### 4.2 Availability of AMFm products versus non-artemisinin therapy and artemisinin monotherapy

Additionally, the AMFm programme aims to displace often used, but older and less effective treatments [chloroquine and sulfadoxine-pyrimethamine (SP)], by making the more trustworthy ACTs widely available for patients [17]. The result obtained shows that this goal is being achieved, as the availability of ACTs via this innovative funding mechanism is helping to reduce the popularity of the older but less-effective antimalarials, like dihydroartemisin, artesunate and chloroquine [2,29]. AMFm AL availability exceeded that of SP, the most common non -artemisinin antimalarial available (86% versus 84%). In some previous surveys carried out in Nigeria, Uganda and Zambia, SP was the most commonly available non-artemisinin antimalarial [2,27-29]. However, a household survey conducted in Nigeria in 2012 showed that caregivers treat more malaria cases in under-five children with non-artemisinin therapy (70%) than ACTs (30%) [31]. More than half of the non-artemisinin therapy involved use of chloroquine. This ought not to be so, as chloroquine has been removed from the National malaria treatment policy as first line treatment for malaria on account of its relative ineffectiveness due to development of resistant strains [16]. Other non-artemisinin treatment availability was also lower than AMFm AL availability, which is as documented elsewhere [30]. The Nigeria unpublished outlet survey of 2012 showed that SP availability was 91% or virtually the same as AMFm AL availability [30]. Like the non-artemisinin therapy, artemisinin monotherapy availability was lower than AMFm ALs in this survey. This is unlike in previous studies, in which dihydroartemisinin was found to be popular [20].

Thus, this study revealed that the popularity of antimalarials like chloroquine (non-artemisinin therapy) and DHA (monoartemisinin therapy) has reduced considerably compared to previous findings. In surveys conducted in 2009 and 2012, chloroquine was the most commonly available non-artemisinin antimalarial [20,31].

### 4.3 Sectoral availability of AMFm products

As shown in previous studies [2,20,29,30], availability was higher in the private sector (pharmacies and patent medicine store) than the public sector (tertiary/secondary hospitals and PHCs); 94% versus 78%, respectively. Availability of AMFm AL products was higher than the innovator brands in most cases. This is similar to a survey carried out in Nigeria in 2012 [30] and another survey on cardiovascular agents conducted in 2010 [32], in which innovator brands were much less available than the generic formulations and more products were found in the private sector than in the public sector (57% to 26%).

### 4.4 RDT availability

Improved availability of RDTs will help guide treatment of fevers that had been presumptively treated as malaria cases [4,5]. RDTs were found in almost a quarter of the facilities in this survey which is better than the 12% recorded in the baseline outlet survey in Nigeria in 2009 [20] and much better than the less than 10% recorded in 2011 surveys conducted in Zambia, Uganda and Nigeria [27-29], although this value is still low. To make a case for a shift from symptom-based diagnosis to parasite-based diagnosis and management of malaria, clinicians require reliable, easy to use and cheap diagnostic tests, like RDTs [22,33].

## 5 Conclusions

Improved availability of ACTs and RDTs was recorded because of the AMFm financing strategy although Artemether/Lumefantrine AMFm products were more widely available than the Artesunate/Amodiaquine AMFm products and RDTs. These findings provide evidence of the effect of national-scale subsidy programmes on drug availability and calls for more government collaboration with the private sector on other health issues affecting communities. Further investigation is required to determine the causes for low values obtained for the Artesunate/Amodiaquine AMFm products and RDTs.
